# Real-world comparative effectiveness of second-line ipilimumab for metastatic melanoma: a population-based cohort study in Ontario, Canada

**DOI:** 10.1186/s12885-020-06798-1

**Published:** 2020-04-15

**Authors:** Wei Fang Dai, Jaclyn M. Beca, Ruth Croxford, Wanrudee Isaranawatchai, Ines B. Menjak, Teresa M. Petrella, Nicole Mittmann, Craig C. Earle, Scott Gavura, Timothy P. Hanna, Kelvin K.W. Chan

**Affiliations:** 1 Cancer Care Ontario, Toronto, ON Canada; 2 Canadian Centre for Applied Research in Cancer Control, Toronto, ON Canada; 3 Institute of Clinical Evaluative Sciences, Toronto, ON Canada; 4 St Michael’s Hospital, Toronto, ON Canada; 5 Institute of Health Policy, Management and Evaluation, University of Toronto, Toronto, ON Canada; 6 Department of Medicine, University of Toronto, Toronto, ON Canada; 7 Sunnybrook Health Sciences Centre, 2075 Bayview Avenue, Toronto, Toronto, Ontario M4N 3M5 Canada; 8 Division of Cancer Care and Epidemiology, Queen’s University Cancer Research Institute, Kingston, Canada; 9 Department of Oncology, Queen’s University, Kingston, ON Canada; 10 Institute of Clinical Evaluative Sciences, Queen’s University, Kingston, ON Canada

**Keywords:** Real-world, Comparative effectiveness, Metastatic melanoma, Immunotherapy

## Abstract

**Background:**

For novel cancer treatments, effectiveness in clinical practice is not always aligned with clinical efficacy results. As such it is important to understand a treatment’s real-world effectiveness. We examined real-world population-based comparative effectiveness of second-line ipilimumab versus non-ipilimumab treatments (chemotherapy or targeted treatments).

**Methods:**

We used a cohort of melanoma patients receiving systemic treatment for advanced disease since April 2005 from Ontario, Canada. Patients were identified from provincial drug databases and the Ontario Cancer Registry who received second-line ipilimumab from 2012 to 2015 (treated) or second-line non-ipilimumab treatment prior to 2012 (historical controls). Historical controls were chosen, to permit the most direct comparison to pivotal trial findings. The cohort was linked to administrative databases to identify baseline characteristics and outcomes. Kaplan-Meier curves and multivariable Cox regression models were used to assess overall survival (OS). Observed potential confounders were adjusted for using inverse probability of treatment weighting (IPTW).

**Results:**

We identified 329 patients with metastatic melanoma (MM) who had received second-line treatments (189 treated; 140 controls). Patients receiving second-line ipilimumab were older (61.7 years vs 55.2 years) compared to historical controls. Median OS were 6.9 (95% CI: 5.4–8.3) and 4.95 (4.3–6.0) months for ipilimumab and controls, respectively. The crude 1-year, 2-year, and 3-year OS probabilities were 34.3% (27–41%), 20.6% (15–27%), and 15.2% (9.6–21%) for ipilimumab and 17.1% (11–23%), 7.1% (2.9–11%), and 4.7% (1.2–8.2%) for controls. Ipilimumab was associated with improved OS (IPTW HR = 0.62; 95% CI: 0.49–0.78; *p* < 0.0001).

**Conclusions:**

This real-world analysis suggests second-line ipilimumab is associated with an improvement in OS for MM patients in routine practice.

## Background

The treatment effects of novel cancer therapies adopted into clinical practice (effectiveness) do not always align with clinical trial outcomes (efficacy) [1]. In particular, for many new cancer drugs, differences between efficacy and effectiveness are often not known, leading to uncertainty in clinical decision-making and drug funding decisions. As such, it is important to investigate the effectiveness of novel systemic therapies in routine practice, using population-based administrative databases, which are reflective of the real-world patient experience.

Ipilimumab, an anti-cytotoxic T-cell lympohocyte-4 monoclonal antibody, was the first immune checkpoint inhibitor therapy to show significant survival benefits in patients with unresectable or metastatic melanoma (MM) [2]. Prior to the introduction of ipilimumab, treatment options for MM patients typically include standard cytotoxic chemotherapy such as dacarbazine or temolozomide. Often, these treatments conferred limited benefit with phase II trial results showing median overall survival around 6.2 months and 1 year survival rate of 25.5% [3]. In Canada, ipilimumab in the second-line setting was the first immunotherapy to receive regulatory approval and was publicly funded in most provinces since 2012. Funding for ipilimumab in Canada was subsequently extended to first-line use in 2015 and to combination therapy with nivolumab in 2019.

In the pivotal trial for ipilimumab, Hodi et al. observed survival benefits (hazard ratio (HR) = 0.66; 95% CI: 0.51–0.87) for patients receiving ipilimumab (median overall survival (OS) = 10.1 months) compared to those receiving glycoprotein 100 peptide vaccine alone (median OS 6.4 months) [2]. Patients receiving ipilimumab had higher survival rates (12 months = 45.6%; 18 months = 33.2%; 24 months = 23.5%) than those receiving glycoprotein100 alone (12 months = 25.3%; 18 months = 16.3%; 24 months = 13.7%). Moreover, the ipilimumab group was observed to maintain long-term benefit, with a 20% survival rate up to the end of the follow up of 54 months. In an analysis of patients with at least two years of potential, 25% of patients in the ipilimumab group survived more than 2 years and 3 years since initial randomization [4]. In the control group 17 and 10% survived more than 2 and 3 years, respectively [4].

To date, only one published study has examined the real-world comparative effectiveness of second-line ipilimumab compared to standard chemotherapy. This study, conducted in Poland, found similar survival benefit as the trial (adjusted HR = 0.65), but observed differences in median OS from the clinical trial experience, including attenuated median OS and less evident survival tail plateau compared to that observed in the trial [2, 5]. Additionally, correction for possible lead-time bias or differences in patient comorbidity between groups was not undertaken and notably the comparison group was contemporary rather than historic. The issue of lead-time bias is especially relevant given that clinical enthusiasm for a new and effective first-in-class drug like ipilimumab could lead to earlier initiation of second line immunotherapy treatment compared to second-line chemotherapy, which could influence observed differences in survival between comparison groups. Moreover, given possible differences in case mix among jurisdictions, it is important to evaluate the real-world comparative effectiveness of second-ipilimumab in a North American population.

Using population-based data from the Canadian province of Ontario, we set out to quantify the real-world effectiveness of second-line ipilimumab in routine practice, as compared to efficacy observed in pivotal trials.

## Methods

We conducted a population-based comparative retrospective cohort study of individuals treated for MM with second-line ipilimumab, chemotherapy, or targeted therapy between 2012 and 2015 in Ontario. Ontario is the largest province in Canada with a population of approximately 14 million people [6]. For patients with MM, the primary treatment is systemic treatments administered by cancer clinics and funded by the provincial government public drug programs.

### Study population

Patients diagnosed with melanoma (International Classification of Disease for Oncology, 3rd edition - topography:C44) who started first-line systemic treatment for advanced melanoma on or after April 1, 2005 were identified from the Activity Level Reporting (ALR) systemic treatment database maintained by the provincial cancer agency, Cancer Care Ontario. The ALR database consists of population-wide systemic treatment records for cancer patients in Ontario. Patients who received first-line treatment with palliative intent were considered for inclusion, defined as the first non-interferon systemic therapy received by the patient. Since a treatment regimen might consist of multiple drugs provided on different days, a line of treatment was defined as consisting of all chemotherapy drugs given to a patient within four days of the start of treatment. The line of treatment was deemed to continue as long as the patient continued to receive at least one of the drugs. The data sources used to identify treatment regimens are presented in Additional file 1.

**Fig. 1:**
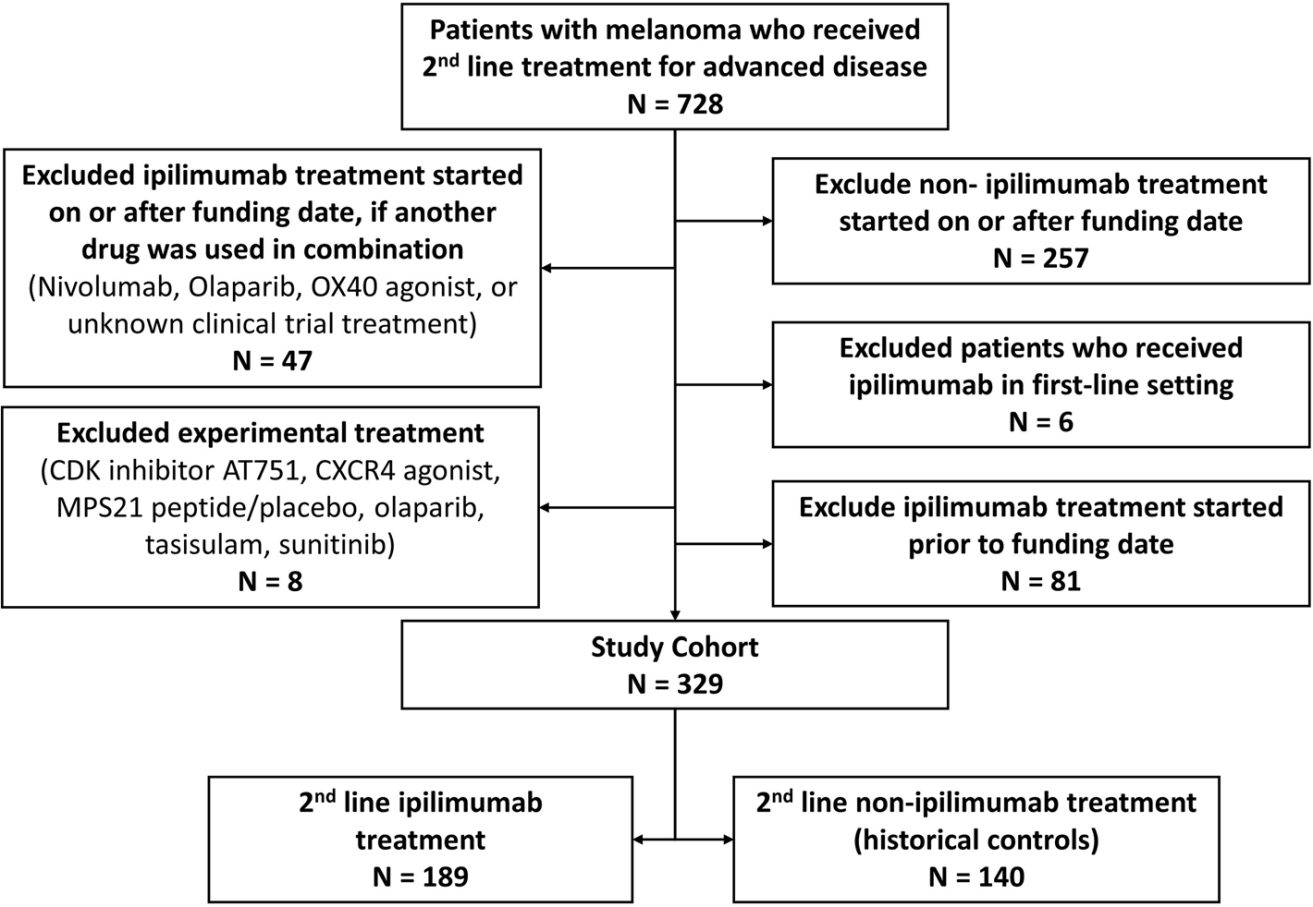
Diagram of study cohort creation

Patients were included in the study if they started a second line of treatment prior to September 13, 2012 (historical controls) or if they started a second line of treatment with ipilimumab between September 13, 2012 and March 31, 2015 (treated). Historical controls were chosen, to permit the most direct comparison to pivotal trial findings. Patients were excluded if they were younger than 18 at the time of their melanoma diagnosis, had been diagnosed with another prior cancer, received ipilimumab as part of their first-line treatment or received experimental treatment/clinical trial agents as part of their 2nd line of treatment. Potential treated patients were excluded if they received ipilimumab in combination with another treatment (Fig. 1). The index date was the date of the start of second-line treatment.

### Outcomes and covariates

The study cohort was linked to administrative databases to determine baseline demographics and date of death. The primary outcome was overall survival, defined as the time from the start of second-line treatment until death. Patients were censored if they emigrated from Ontario or remained alive at the end of follow up on March 31, 2017. Patient demographic characteristics including age, sex, postal code, and date of death were obtained from the Registered Persons Database. Patient comorbidity was calculated using a weighted average of the Adjusted Clinical Groups (Johns Hopkins ACG(R) System, Version 10) which were based on diagnoses obtained from hospital discharge records and physician claims records in the 2 years prior to the index date, excluding cancer diagnoses [7]. In addition, the Charlson score was calculated based on diagnoses found in hospital discharge records in the 2 previous years, excluding cancer diagnoses [8]. Statistics Canada’s 2016 census data was used to obtain rural/urban status of the patient’s residence and neighborhood income quintile based on postal codes. Additional information on other systemic therapy and radiotherapy was obtained from the ALR and hospital discharge records. These datasets were linked using unique, encoded identifiers and analyzed at ICES.

### Statistical analysis

For patient and treatment characteristics, mean and standard deviation or median and interquartile range were reported for continuous variables, and frequencies and percentages were calculated for categorical variables. Baseline characteristics of the ipilimumab-treated and control patients were compared using chi-square tests for binary covariates, Kruskal-Wallis tests for categorical variables, t-tests for normally distributed continuous variables, and Wilcoxon rank sum tests for skewed numerical variables.

To adjust for differences between groups, propensity scores were calculated using logistic regression, and were used to calculate inverse probability of treatment weights (IPTW) [9]. Baseline covariates used to calculate the final propensity scores were patient age, place of residence, the comorbidity score and Charlson score, prior radiation to the brain, prior radiation to other body parts, and time from diagnosis to the start of second line treatment. Standardized differences for the IPTW-weighted covariates were calculated. Standardized differences ≤0.10 are generally considered to represent acceptable balance [9].

Overall survival was assessed using Kaplan-Meier method and log-rank test was performed to assess the difference between groups. Time to death between groups was also compared using a Cox proportional hazards model, weighting patients in each group by their IPTWs. Due to the use of IPTWs, the resulting hazard ratios estimate the average treatment effect, an estimate of the treatment effect if ipilimumab rather than the historical treatments were used as second line treatment in the population of melanoma patients who are similar to those included in this study. Sensitivity analyses were performed, incorporating third-line treatment (if any) into the survival analyses by i) adjusting for the use of third-line treatment as a time-varying confounder; ii) adjusting third-line checkpoint inhibitors treatment as a time-varying confounder; iii) censoring patients at the time of initiating third-line treatment; and iv) excluding patients who received third-line treatment.

Two-sided *p*-values < 0.05 were considered to be statistically significant. SAS version 9.4 (SAS institute, Cary, North Carolina) was used for the analyses.

## Results

### Study population

A total of 728 patients received a second-line treatment for MM between September 13th, 2008 and March 31st, 2015. Of these identified patients, 329 patients met eligibility criteria and were included (Fig. 1). The final cohorts consist of 140 (42.6%) control patients who received second-line treatment with chemotherapy or targeted therapy prior to the ipilimumab funding date and 189 (57.4%) treated patients who received second-line ipilimumab treatment after the funding date.

### Baseline characteristics

Baseline demographic and clinical characteristics are presented in Table 1. In the total unweighted cohort, the mean age is 58.9 (SD: 16.4) years, 67.8% were males, and 87.5% lived in urban regions. At baseline, patients who are treated with second-line ipilimumab were older (61.7 years vs 55.2 years) compared to historical controls. There were no significant differences in sex, income quintile, comorbidities, and prior receipt of radiation between the ipilimumab-treated and the historical control patients.

**Table 1:** Baseline Characteristics

Variables	Unweighted			IPTW Weighted		
	Historical Controls *N* = 140	2nd line Ipilimumab *N* = 189	*P*-value	Historical Controls *N* = 288.4	2nd line Ipilimumab *N* = 311.2	Weighted standardized difference
Age at second line (years), mean (SD)	55.2 (12.7)	61.7 (13.6)	<.001	57.5 (18.5)	59.5 (17.9)	0.11
Males, n (%)	96 (68.6)	127 (67.2)	0.79	66.63%	67.71%	0.02
Income quintile, n (%)			0.41			
Lowest	18 (12.9)	20 (10.6)		11.47%	10.39%	0.03
Medium to Low	21 (15.0)	28 (14.8)		15.73%	16.47%	0.02
Medium	37 (26.4)	37 (19.6)		26.00%	16.99%	0.22
Medium to High	28 (20.0)	52 (27.5)		19.88%	26.89%	0.17
Highest	36 (25.7)	52 (27.5)		26.93%	29.26%	0.05
Urban, n (%)	121 (86.4)	167 (88.4)	0.60	88.53%	89.12%	0.02
Austin morbidity score, mean (SD)	15.9 (11.3)	16.5 (11.9)	0.63	15.99 (16.8)	16.1 (15.4)	0.01
Number of ADGs, median (IQR)	8 (6–10)	9 (6–11)	0.21	8.3 (4.5)	8.5 (4.4)	0.04
Charlsons score, n (%)			0.15			
0	123 (87.9)	149 (78.8)		83.5%	82.5%	0.03
1+	17 (12.1)	40 (21.2)		16.5%	17.5%	0.08
Prior radiation (any), n (%)	79 (56.4)	105 (55.6)	0.88	55.40%	56.00%	0.01
Prior radiation of brain, n (%)	30 (21.4)	46 (24.3)	0.54	22.37%	22.98%	0.01
Prior radiation other than brain, n (%)	65 (46.4)	74 (39.2)	0.19	45.04%	40.20%	0.1
Prior resection (any), n (%)	102 (72.9)	139 (73.5)	0.89	55.40%	56.00%	0.01
Prior brain resection, n (%)	<=5	13 (6.9)	0.19	4.99%	6.63%	0.07
Prior other resection, n (%)	12 (8.6)	9 (4.8)	0.16	6.69%	6.68%	0
Time from diagnosis to start of second line (months), median (IQR)	32.5 (11.9–57.5)	18.0 (8.4–38.5)	0.007	39.9	42.9	0.04
Time from end of first-line treatment to start of second-line treatment (months), median (IQR)	1.7 (1.0–3.3)	1.0 (0.7–1.7)	< 0.001	1.8	2.6	0.19
Time from diagnosis to start of first line (months), median (IQR)	23.7 (6.5–54.1)	12.6 (4.1–33.7)	< 0.001	35.1	36.9	0.03
First-line treatment, n (%)			< 0.001			
Chemotherapy	110 (78.6)	119 (63.0)		81.7%	58.4%	0.53
BRAF/MEK	13 (9.3)	59 (32.3)		9.7%	36.9%	0.68
Non-ipilimumab Immunotherapy	8 (5.7)	8 (4.2)		5.2%	4.0%	0.06
Others	9 (6.4)	<= 5		3.5%	0.7%	0.19

*Percentage shown in the table is column %

Abbreviation: *SD* Standard Deviation, *IQR* Interquartile range, *ADG* Adjusted Diagnosis Groups;

The median time from diagnosis to initiating second-line treatment was 18 months (95%CI: 8.4–38.5 months) for second-line ipilimumab patients and 32.5 months (95% CI: 11.9–57.5 months) for historical controls (Table 1). The median time between the end of first-line and the start of second-line treatment was shorter for patients receiving second-line ipilimumab (1 month vs 1.7 months; *p*-value < 0.001) (Table 3). The majority of the historical controls received first-line chemotherapy (78.6%), while a minority received first-line BRAF/MEK (9.3%), with the remainder (10%) receiving either non-ipilimumab immunotherapy or other treatments for first-line therapy. The majority of patients who received second-line ipilimumab (treated) received chemotherapy (63%) or BRAF/MEK inhibitors (32.3%) as their first-line treatment.

Weighted standardized difference between the treated and historical controls for all baseline characteristics were calculated after IPTW adjustment. All standardized differences were less than 0.1 with the exception of age, income quintile (medium and medium to high), and time from end of first-line treatment to start of second-line treatment.

### Treatment patterns

Approximately half (49.2%) of the patients receiving second-line ipilimumab completed all four planned doses of ipilimumab; the remaining 14.8% had one dose, 19.6% had 2 doses, and 13.2% had 3 doses. Amongst the historical controls, 127 patients received chemotherapy (e.g. dacarbazine and temozolomide) and other treatment (e.g. tyrosine kinase inhibitors), while 13 patients received BRAF/MEK (e.g. vemurafenib, dabrafenib). Amongst the study cohort of patients receiving second-line treatments, 38 (27.1%) historical controls and 64 (35.5%) ipilimumab patients proceeded to receive at least one third-line treatment. Of those patients who received third-line treatments, 27 (71%) historical controls and 51 (76.1%) ipilimumab patients received immunotherapy, while the remaining patients received chemotherapy or other treatments. Amongst the historical controls, the third-line immunotherapy received were mainly ipilimumab whereas the immunotherapy received by the cases were either nivolumab or pembrolizumab.

### Overall survival

**Fig. 2:**
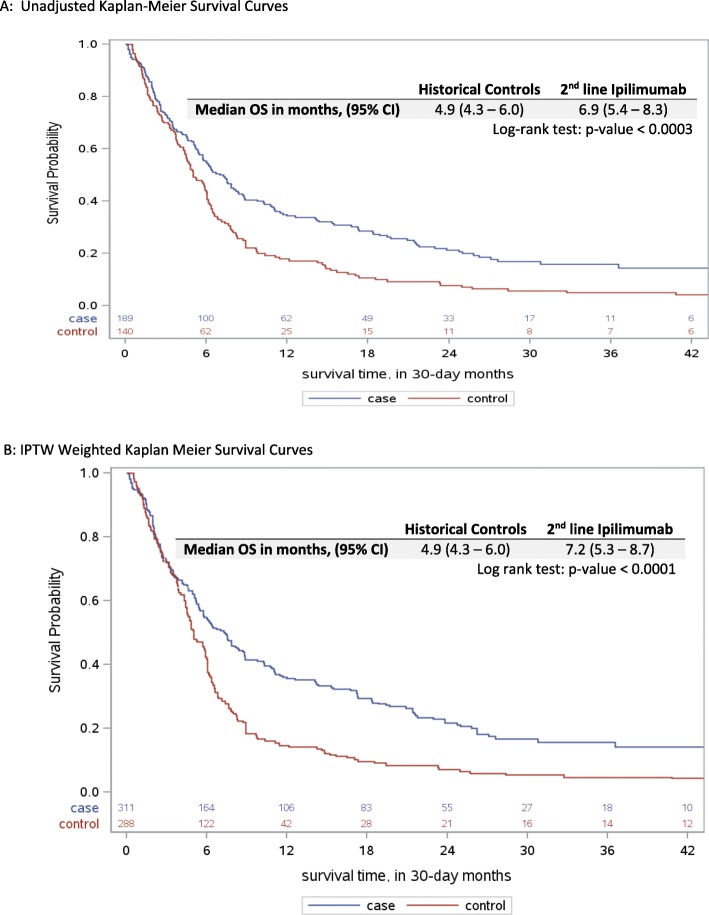
Overall survival with 2nd line ipilimumab and historical controls (**a**) Unadjusted (**b**) IPTW weighted

The cohort of patients were followed up until March 31st, 2017 with a median follow-up of 30.4 months (95% CI: 27.9–37.7 months) in second-line ipilimumab patients and 71.2 months (95% CI: 70.3–116.5 months) in historical controls (Table 2). Crude median OS was 6.9 months (95% CI: 5.4–8.3 months) and 4.9 months (95% CI: 4.3–6.0 months) for patients receiving second-line ipilimumab and historical controls, respectively (Fig. 2a). The adjusted median OS is also greater in second-line ipilimumab (7.2 months; 95% CI: 5.3–8.7 months) compared to historical controls (4.9 months; 95% CI: 4.3–6.0 months). OS was significantly improved for patients receiving second-line ipilimumab after IPTW adjustment (*p*-value < 0.0001) (Fig. 2b).

**Table 2:** Survival Outcomes

	Historical Controls *N* = 140	2nd line Ipilimumab *N* = 189
Median follow-up months, (95% CI)	30.4 (27.9–37.3)	71.2 (70.3–116.5)
1-year survival rates, (95% CI)		
Unadjusted	17.1% (11–23%)	34.3% (27–41%)
IPTW-adjusted	17.1% (11–23%)	35.6% (27–43%)
2-year survival rates, (95% CI)		
Unadjusted	7.1% (2.9–11%)	20.6% (15–27%)
IPTW-adjusted	7.1% (2.9–11%)	21.1% (14–28%)
3-year survival rates, (95% CI)		
Unadjusted	4.7% (1.2–8.2%)	15.2% (9.6–21%)
IPTW-adjusted	4.7% (1.2–8.2%)	14.3% (8.0–21%)

Survival was higher at 1-year, 2-year, and 3-year for the ipilimumab group compared to controls in both the unadjusted and IPTW-adjusted analysis (Table 2). Moreover, we observed sustained benefit in the 3 year OS in the ipilimumab group (IPTW-adjusted: 14.3%; 95% CI: 8–21%). Second-line ipilimumab is significantly associated with improved OS in the unadjusted and IPTW weighted Cox proportional hazard model (Unadjusted HR = 0.65; 95% CI: 0.52–0.82; IPTW HR = 0.62; 95% CI: 0.52–0.73) (Table 3).

**Table 3:** Hazard Ratio for Overall Survival & Sensitivity Analysis (2nd line ipilimumab vs historical controls)

		Hazard Ratio (95% CI)	*P*-value
**Primary Analyses**	Model A: Unadjusted Model	0.65 (0.52–0.82)	0.0003
	Model B: IPTW Weighted Model	0.62 (0.52–0.73)	< 0.0001
**Sensitivity Analyses**	Model C: IPTW Weighted Model adjusting for 3rd line	0.64 (0.53–0.76)	< 0.0001
	Model D: IPTW Weighted Model adjusting for 3rd line checkpoint inhibitor treatment		
	2nd line Ipilimumab	0.63 (0.53–0.75)	< 0.0001
	Historical Controls	Ref	
	Model E: Censoring patients at start of 3rd line		< 0.0001
	2nd line Ipilimumab	0.60 (0.48–0.73)	
	Historical Controls	Ref	
	Model F: Excluding patients who started 3rd line treatment		0.0001
	2nd line Ipilimumab (*n* = 122)	0.67 (0.55–0.81)	
	Historical Controls (*n* = 102)	Ref	

### Sensitivity analysis

Sensitivity analyses were conducted to explore the effect of third line treatment on the association between second-line ipilimumab and OS (Table 3 Models C-F). Despite increasingly conservative assumptions, the estimated ipilimumab treatment effect remained largely unchanged. An interaction term between second-line ipilimumab and third-line treatment, added to models C and D, was not significant (p_interaction_ = 0.085 and 0.12, respectively). Among those patients who received second line ipilimumab and started on third line treatment, the choice of treatment was not significantly associated with survival (immunotherapy vs. other treatment: HR = 0.75; 95% CI: 0.46–1.22 p = 0.25).

## Discussion

In our real-world population-based study, we observed improved survival associated with second-line ipilimumab compared with second-line chemotherapy or targeted therapy (median OS 6.9 vs 4.9 months; Unadjusted HR = 0.65; Adjusted HR = 0.62). The estimated treatment effect was robust under different methods to adjust for the effect of subsequent treatment (HR range = 0.63–0.67). Moreover, despite more older patients receiving second-line ipilimumab (mean age: 61.7 years) compared to chemotherapy or targeted treatment (mean age: 55.2 years) in the real-world, the observed relative treatment effect was still consistent with those reported in the pivotal trial (HR = 0.66; 95% CI: 0.51–0.87) [2]. Additionally, we observed approximately half of the second-line ipilimumab patients completed the planned four doses. This was slightly lower than the 64.2% of patients who received all doses in the trial [2].

In this study, the hazard ratio for overall survival (Unadjusted HR = 0.65; Adjusted HR = 0.62) was similar to the pivotal trial (HR = 0.66). The survival curve also plateaued around two to three years, similar to the pivotal trial. In contrast, the relative estimate of survival benefit, median OS, was shorter in the real-world compared to the pivotal trial for both the ipilimumab patients and the control patients. In particular, the real-world median OS was 2.9 months shorter (Real-world: 7.2 months; RCT: 10.1 months) in the ipilimumab group and 1.5 months shorter in the control group (Real-world: 4.9 months; RCT: 6.4 months), compared to clinical trial outcomes. As such, the magnitude of benefit for OS observed in the trial was approximately 3.7 months whereas the real-world effectiveness was 2.3 months. In our real-world setting, less ipilimumab patients were alive at 2-years (Real-world: 21.1%; RCT: 25%) and 3-years (Real-world: 14.3%; RCT: 25%) [4]. Similarly, less control patients were alive at 2-years (Real-world: 7.1%; RCT: 17%) and 3-years (Real-world: 4.7%; RCT: 10%) [4]. This difference may be attributable older patients receiving second-line ipilimumab in the real-world as compared to the trial (mean age: 61.7 vs 56.8 years). Moreover, 10% of treated and 14% of controls in the trial had central nervous system (CNS) metastases at baseline and received previous treatments for it. In our real-world study, a little over 20% of patients in each group had radiation to the brain prior to index systemic treatment, which may have affected absolute survival estimates.

In the population-based study conducted by Polkowska et al., which also compared second-line ipilimumab to second-line chemotherapy, the OS and HR of second-line ipilimumab was similar to our study and the trial. While the median OS were similar between the controls in both studies, patients receiving second-line ipilimumab in our study (median OS: 7.2; 95% CI: 5.3–8.7) had a slightly higher median OS than those patients in Poland (median OS: 5.9; 95% CI: 5.6–8.4), though the confidence intervals were overlapping between the two studies. Additionally, difference in median OS may also be affected by the difference in access to treatments. The authors mentioned that immunotherapies are only available for second and subsequent lines of treatment. While first-line immunotherapies were not publicly funded in Ontario at the time of our cohorts, around 5% of patients in each group had received non-ipilimumab immunotherapies from clinical trials or private payers. Furthermore, the third-line treatment availability may also differ between the two studies. In our study, controls were more likely to receive third-line ipilimumab while treated were more likely to receive third-line nivolumab or pembrolizumab. Despite the differences, both observed median OS for second-line ipilimumab was within the range observed in some of the single-arm studies, which ranged between 6.4 to 8.8 months [10–14].

Our study has a number of notable strengths. We had an extensive collection of linked data, providing detailed information on patient characteristics and treatments for an entire population. Thus, we were able to adjust for many more potential confounders using propensity score methods in contrast to other population-based studies. These variables include rurality, socioeconomic status, previous resection, comorbidity status, and time from diagnosis to initiating second-line treatment. Additionally, we also used different sensitivity analyses to explore the effect of subsequent third-line treatment given that real-world data are non-randomized and that subsequent therapies can be a potential source of confounding. The survival benefit of second-line ipilimumab persisted after these adjustments.

Our study has several limitations. First, inherent to observational studies, our estimates may have been affected by residual confounding from unbalanced or unmeasured variables such as performance status, lactate dehydrogenase, and body mass index. Our analysis did not adjust for performance status, though we were able to control for differences in comorbidity, which could affect functional status. Additionally, given this is a pre-funding vs post-funding comparison, it is unlikely that the distribution of performance status of the population would change significantly. Data on lactate dehydrogenase (LDH) was also not available, though notably in the pivotal trial by Hodi et al., there was no significant difference in ipilimumab treatment effect based on LDH [2], and it is unlikely that there would be imbalances given the pre/post design with nearly all patients receiving ipilimumab after funding. Second, in contrast to the randomized trial, the comparator in our study consists of historical controls, since once second-line ipilimumab became available, a very small number of patients received chemotherapy or targeted therapy. While historical controls avoid the small sample size of controls or confounding by indication after ipilimumab funding, historical comparators might be confounded by secular trends such as changes in clinical practice (e.g. radiation or resection practices for metastases, availability of other systemic therapies (see Additional file 1 for public funding timeline) that might bias in favor of ipilimumab. Lastly, after second-line ipilimumab was funded, some patients may have truncated their first-line treatment to access ipilimumab for second-line treatment as soon as possible, as there would have had been no option for first-line immunotherapy. This funding change may have resulted in differences in the extent of disease at the start of second line treatment between treatment groups. Drysdale et al. shown that 40% of patients who received first-line ipilimumab for MM had received prior chemotherapies for less than 60 days during a similar study period in Ontario [15]. Time between diagnosis to second-line treatment was adjusted for via the propensity score method to address potential bias, though residual imbalance remained in our study.

In addition to validating the efficacy observed in clinical trials, our study also informs policy decisions in Ontario. While ipilimumab was the first immunotherapy to enter the treatment landscape, other immunotherapies, PD1 inhibitors pembrolizumab and nivolumab, have become available in both first-line and second-line setting in Ontario, along with nivolumab in combination with ipilumumab [16–19]. Despite the availabilities of these therapies, the effectiveness of second-line ipilimumab is still of importance for patients who may not be able to tolerate aggressive immunotherapy or progress on combination targeted treatments. Potential future area of investigation can examine the effectiveness of second-line ipilimumab after first-line PD-1 inhibitors and the comparative effectiveness between ipilimumab monotherapy and combination therapies. Additional future research relevant for policy decision includes the comparative toxicity of second-line ipilimumab and the effect of immunotherapy on patient symptoms, especially for patients who experience metastases to other body regions at baseline.

## Conclusion

Overall, our findings illustrate the real-world comparative effectiveness of second-line ipilimumab, which was associated with improved survival in patients with MM compared to historical controls, and with HRs similar to those observed in the pivotal trial and other population-based studies. While the relative treatment effect was similar to the pivotal trial, the median overall survival absolute treatment effect observed among real-world patients treated with ipilimumab was smaller and similar to that observed in the other population-based studies.

## Supplementary information

**Additional file 1: Appendix:** Figure 1: Funding timeline of metastatic melanoma treatments in Ontario. Table 1: Summary of administrative databases.

## Data Availability

The data that support the findings of this study are available from ICES, but restrictions apply to the availability of these data, which were used under license for the current study, and so are not publicly available. Data are however available from the authors upon reasonable request and with permission of ICES.

## Abbreviations

MM: Metastatic melanoma
HR: Hazard ratio
OS: Overall survival
ALR: Activity Level Reporting
ACG: Adjusted Clinical Group
IPTW: Inverse Probability Treatment Weighting
SD: Standard deviation
IQR: Interquartile Range
CI: Confidence Interval

## References

[1] Lakdawalla DN, Shafrin J, Hou N, et al. Predicting real-world effectiveness of Cancer therapies using overall survival and progression-free survival from clinical trials: empirical evidence for the ASCO value framework. Value Heal. 2017. 10.1016/j.jval.2017.04.003. 10.1016/j.jval.2017.04.003 28712615

[2] Hodi FS, O’Day SJ, McDermott DF, et al. Improved survival with ipilimumab in patients with metastatic melanoma. N Engl J Med. 2010. 10.1056/NEJMoa1003466. 10.1056/NEJMoa1003466 PMC3549297 20525992

[3] Korn EL, Liu PY, Lee SJ, et al. Meta-analysis of phase II cooperative group trials in metastatic stage IV melanoma to determine progression-free and overall survival benchmarks for future phase II trials. J Clin Oncol. 2008. 10.1200/JCO.2007.12.7837. 10.1200/JCO.2007.12.7837 18235113

[4] McDermott D, Haanen J, Chen TT, Lorigan P, O’Day S. Efficacy and safety of ipilimumab in metastatic melanoma patients surviving more than 2 years following treatment in a phase III trial (MDX010-20). Ann Oncol. 2013. 10.1093/annonc/mdt291. 10.1093/annonc/mdt291 23942774

[5] Polkowska M, Ekk-Cierniakowski P, Czepielewska E, Wysoczański W, Matusewicz W, Kozłowska-Wojciechowska M. Survival of melanoma patients treated with novel drugs: retrospective analysis of real-world data. J Cancer Res Clin Oncol. 2017. 10.1007/s00432-017-2453-z. 10.1007/s00432-017-2453-z PMC11818956 28608286

[6] Canada S (2018). Annual demographic estimates : Canada.

[7] Austin PC, Van Walraven C. The mortality risk score and the ADG score: two points-based scoring systems for the Johns Hopkins aggregated diagnosis groups to predict mortality in a general adult population cohort in Ontario, Canada. Med Care. 2011. 10.1097/MLR.0b013e318229360e. 10.1097/MLR.0b013e318229360e PMC4617828 21921849

[8] Sundararajan V, Henderson T, Perry C, Muggivan A, Quan H, Ghali WA. New ICD-10 version of the Charlson comorbidity index predicted in-hospital mortality. J Clin Epidemiol. 2004. 10.1016/j.jclinepi.2004.03.012. 10.1016/j.jclinepi.2004.03.012 15617955

[9] Austin PC. An introduction to propensity score methods for reducing the effects of confounding in observational studies. Multivariate Behav Res. 2011. 10.1080/00273171.2011.568786. 10.1080/00273171.2011.568786 PMC3144483 21818162

[10] Jochems A, Leeneman B, Franken MG, et al. Real-world use, safety, and survival of ipilimumab in metastatic cutaneous melanoma in the Netherlands. Anti-Cancer Drugs. 2018. 10.1097/CAD.0000000000000629. 10.1097/CAD.0000000000000629 29659371

[11] Russi A, Damuzzo V, Chiumente M, et al. Ipilimumab in real-world clinical practice: efficacy and safety data from a multicenter observational study. J Chemother. 2017. 10.1080/1120009X.2017.1311444. 10.1080/1120009X.2017.1311444 28398170

[12] Middleton MR, Dalle S, Claveau J, et al. Real-world treatment practice in patients with advanced melanoma in the era before ipilimumab: results from the IMAGE study. Cancer Med. 2016. 10.1002/cam4.717. 10.1002/cam4.717 PMC4944869 27118102

[13] Khoja L, Atenafu EG, Ye Q, et al. Real-world efficacy, toxicity and clinical management of ipilimumab treatment in metastatic melanoma. Oncol Lett. 2016. 10.3892/ol.2015.4069. 10.3892/ol.2015.4069 PMC4734313 26893783

[14] Krajsová I, Arenberger P, Lakomý R, et al. Long-term survival with ipilimumab: experience from a national expanded access program for patients with melanoma. Anticancer Res. 2015;35(11):6303–10. 26504067

[15] Drysdale E, Peng Y, Nguyen P, Baetz T, Hanna TP. A population-based study of the treatment effect of first-line ipilimumab for metastatic or unresectable melanoma. Melanoma Res. 2019. 10.1097/CMR.0000000000000582. 10.1097/CMR.0000000000000582 PMC6887627 30789386

[16] Ribas A, Puzanov I, Dummer R, et al. Pembrolizumab versus investigator-choice chemotherapy for ipilimumab-refractory melanoma (KEYNOTE-002): a randomised, controlled, phase 2 trial. Lancet Oncol. 2015. 10.1016/S1470-2045(15)00083-2. 10.1016/S1470-2045(15)00083-2 PMC9004487 26115796

[17] Robert C, Schachter J, Long GV, et al. Pembrolizumab versus Ipilimumab in Advanced Melanoma. N Engl J Med. 2015. 10.1056/NEJMoa1503093. 10.1056/NEJMoa1503093 25891173

[18] Robert C, Long GV, Brady B, et al. Nivolumab in previously untreated melanoma without BRAF mutation. N Engl J Med. 2015. 10.1056/NEJMoa1412082. 10.1056/NEJMoa1412082 25399552

[19] Hodi FS, Chesney J, Pavlick AC, et al. Combined nivolumab and ipilimumab versus ipilimumab alone in patients with advanced melanoma: 2-year overall survival outcomes in a multicentre, randomised, controlled, phase 2 trial. Lancet Oncol. 2016. 10.1016/S1470-2045(16)30366-7. 10.1016/S1470-2045(16)30366-7 PMC5630525 27622997

