# Supplementary material for: Real-world comparative effectiveness of second-line ipilimumab for metastatic melanoma: a population-based cohort study in Ontario, Canada
Source: BMC Cancer. 2020 Apr 15;20:304. doi: 10.1186/s12885-020-06798-1 (PMC7158109; doi:10.1186/s12885-020-06798-1)
Supplement: Supplementary file 1 — Additional file 1: Appendix: Figure 1: Funding timeline of metastatic melanoma treatments in Ontario. Table 1: Summary of administrative databases. [file 12885_2020_6798_MOESM1_ESM.docx]

**Appendix**

**Appendix: Tables and Figures**

Figure 1: Funding timeline of metastatic melanoma treatments in Ontario


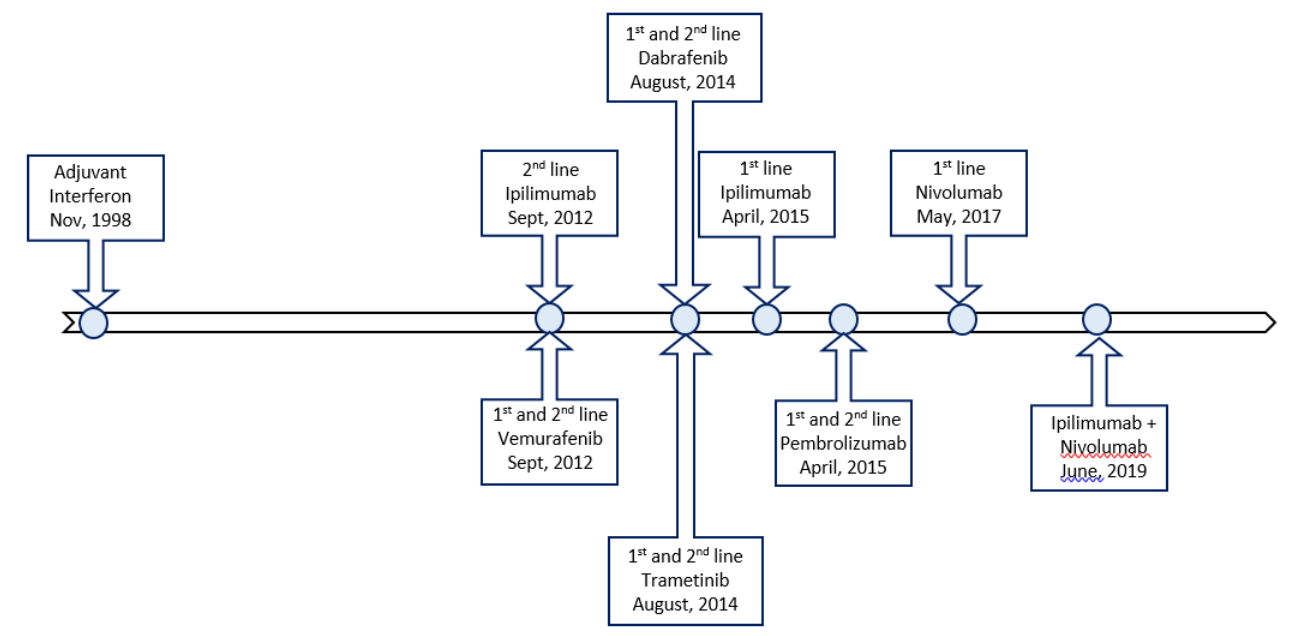


| **Table 1: Summary of administrative databases**  Ontario residents are covered by single-payer health care insurance (the Ontario Health Insurance Plan) which covers the costs of all medically necessary services. Details of insured services, including cost information, are found in a number of administrative databases. The major databases used for the current study are briefly described below. | | |
| --- | --- | --- |
| Database | Acronym | Description |
| Registered Persons Database | RPDB | Includes basic demographic information on anyone who has ever received an Ontario health card number, including date of birth, sex, and date of death (if applicable). The database also contains postal code information, which is linked to Statistics Canada census data to obtain neighborhood income quintile and health region. The RPDB records are supplemented at ICES with each person’s date of last contact with the healthcare system. |
| Ontario Cancer Registry | OCR | Includes a record of all primary cancers identified in Ontario residents. In Ontario, it is mandatory to report cancer diagnoses to Cancer Care Ontario for inclusion in the OCR. The registry contains the date of diagnosis and details of the diagnosis. |
| Activity Level Reporting | ALR | The systemic dataset within the ALR provides visit-level information on systemic treatments, including visit date and the chemotherapy and supportive drugs administered. Receipt of radiation therapy is documented in the radiation dataset and provides visit level information including the body part irradiated. |
| Discharge Abstract Database | CIHI-DAD | Comprised of information on all inpatient hospital stays, including diagnoses and procedures. Reporting of inpatient hospital stays is mandatory in Ontario. . |
| Ontario Health Insurance Plan Physician Claims Database | OHIP | Comprised of all physician and laboratory claims for services covered by OHIP, including a fee code for the service provided, a diagnosis code, the physician specialty, and date of visit. |
| Ontario Drug Benefits | ODB | Comprised of claims derived from pharmacists distributing a medication on the ODB formulary to any eligible Ontario residents (≥65 years old, ≤24 years old, residents of long-term care facilities/homes for special care, people receiving services under the Home Care Program, Trillium Drug program recipients, people receiving social assistance, and people eligible for the special drugs programs). Contains medication details and date. |
| National Ambulatory Care Reporting System | NACRS | Comprised of information from outpatient care: same day surgery, emergency department visits and visits to out-patient hospital clinics (cancer clinic, dialysis). Each record specifies procedures and diagnoses relevant to the visit. |
| New Drug Funding Program | NDFP | Comprised of claims for anti-cancer therapies appearing on the NDFP formulary, a publicly-funded drug program through Cancer Care Ontario. Each record specifies the medication received and date of the visit. |
